# 

**Published:** 1902-01

**Authors:** 


### CONTENTS.

ORIGINAL ARTICLES:
Reply of Professor E. Nocard to Professor Koch . . . . . . . . . . . 1
Municipal Meat-inspection Legislation. By G. R. White, D.V.S. . . . . . . 6
A New Plan for Controlling Tuberculosis of Cattle. By Columella . . . . . 16
President's Address, Illinois State Veterinary Medical Association . . . . . 21
A Contrivance for the Ready Handling of Disabled Horses. By Frederick Griffith . 25
Trouble on the Farm. By Holman F. Day . . . . . . . . . . 26

DEPARTMENT OF SURGERY:
Hoof Diseases, *continued*—The Points of a Draught-horse . . . . . . . . 28

ABSTRACTS FROM FOREIGN JOURNALS . . . . . . . . . . . . . . . 32

REPORTS OF CASES:

An Enzoötic Attack of Chorea Among Cattle. By A. D. Knowles, V.S. . . . 37

Polyhydramnios, or Dropsy of the Amnion. By V. P. Smith, V.S. . . . . . 40

EDITORIALS:

Where in 1902? . . . . . . . . . . . . . 42

Editorial Strabismus . . . . . . . . . . . . 42

A Plan for the Disposal of Tuberculous Animals . . . . . . . 43

"December's Garnering" . . . . . . . . . . . 43

COMMENTS ON THE DEATH OF DR. HUIDEKOPER . . . . . . . . . 44

NECROLOGY . . . . . . . . . . . . . . . . . 46

ASSOCIATION PROCEEDINGS—COMMUNICATION—CORRESPONDENCE—SELECTIONS—AMONG THE COLLEGES—SOCIETY PROCEEDINGS—NOTES—PERSONALS . . 47-62

